# Domestic Summary

**Published:** 1839-05-18

**Authors:** 


### DOMESTIC SUMMARY.

*Professional Charges. Autopsy for Legal Purposes. Letters of Consultation.*—At its last session, the Medical Society of Augusta, passed, with unanimous vote, a resolution, that autopsic examinations, for legal purposes, are proper subjects of charge, and that the price for each and every such examination, should be from twenty-five to one hundred dollars.

The principal arguments on which this resolution was based, were, 1st. That the County is always able to pay, as well for professional labour and service for its own purposes, as for any other commodity. 2d. That a man's professional qualifications, whereby he is rendered competent to the discharge of this service, are his capital, or his stock in trade, in a pecuniary point of view, and neither the public, nor any individual, has a right to their use, without a satisfactory return for the same, any more than the public or any individual, has to take, without due remuneration, the labour or property of any other man; and 3d. That these anatomical investigations demand for themselves an ability, which is found only in ample scientific attainments—that they require much time for their proper performance, and much more in attendance on the legal investigations to which they lead, and to which they are all-important. To these we may add, a peculiar weight of responsibility; because on medical, more than any other testimony in such cases, a correct decision depends, in which human life is concerned. By the slightest error, or delinquency in the discharge of this duty, the guilty may be allowed to escape the just inflictions of the law, or the innocent caused to suffer the punishment due only to the guilty.

It may be an inquiry in the mind of some, whether such a resolution as that alluded to in the beginning of this lecture is, or ever would be, called for—whether any counter sentiment on the subject exists, to call forth this action of the medical community? To this inquiry we reply, that a case is now pending in the Superior Court of this County, in consequence of the refusal, on the part of the County, to afford to the anatomist in such a case, a satisfactory remuneration for his services. In this movement the members of the Society feel themselves sustained by the laws of the land, as fairly as they would be under an attempt on the part of the general government to force them to the *gratuitous* discharge of the duties of an army surgeon.

The task is, as we have said, a most disagreeable one; and one which it is the desire of most practitioners to avoid. In case of refusal to discharge those duties according to the demands of justice, the law may, and should exercise compulsory powers; but not without ample remuneration.

Another subject of charge for professional services, than which there is none more proper, but which has been overlooked alike by the profession and the community in this section, is consultation by letter. It is a frequent occurrence for a physician to receive a letter from some other practitioner, or a patient at a distance, making a large demand on his time, attention, and labour—no fee inclosed, nor even the postage paid. It would seem, from such communications, that the writer of such letter presumed on the sufficiency of the *compliment* thus paid to a physician, for paying the debts of the latter, or bearing the current expenses of his family, and paying the postage to boot. Truly, honour is pleasant enough to most men, but he who has learned that to live honestly and honourably, the necessary expenses of life, and of the profession, must be met with promptness, will say, "honour to the dogs," in such a case.

It is worse than useless, to open accounts with persons at a distance of one hundred or more miles; as the collection would cost more than it would profit. It is, therefore, indispensable, that a fee fully sufficient to reward the physician for his labour in the premises, should accompany the letter, or application otherwise made, or a certificate of the disability of the patient to reward the adviser; for, otherwise, a a prompt return, (if return at all,) cannot be reasonably expected. Physicians who are most frequently consulted in this way, have generally enjoyed the *honour* of high confidence before, and for a length of time—therefore, a mere expression of confidence is no reward. Such persons are, moreover, not "gentlemen of leisure," but are busy men, replete with important engagements; such as are not, are rarely worthy of such consultations; for it is by their habitual professional engagements and studious habits, that they obtain that worth in the profession which causes their advice to be thus sought. To men thus engaged, *time* is more precious than gold. Time, and mental, and coporeal labour are their capital, and it is not more just and reasonable, to expect such men to take these, their capital, from a profitable employment, and bestow them gratuitously, than it would be to expect or ask a monied institution, to draw its capital from those employments by which it *returns, with interest*, and give it to an individual whom it is never expected will be heard of again. No. If advice sought in this way be necessary and proper at all, it is only on the *present state* of the case described. The advice must, therefore, in order to be productive of good, be *prompt*. Nothing, therefore, can generally secure this promptness, but *a competent fee in hand*, or a clear and fair claim on the charity of the physician; which latter, to the honour of the profession, is seldom slighted.—*Southern Med. and Surg. Journal.*

*Case of Expulsion of a Fœtus after the death of its Mother.* By J. C. Nott, M.D. of Mobile.—This patient was a negro woman, aged about 35, belonging to Robert Purvis, merchant of Mobile. She had menstruated irregularly for twelve months before conception, and during this time her health was not good. I was called first to see her about the middle period of pregnancy—she was then complaining of pains in the uterus, which became more troublesome as gestation advanced. During the last month, the pains were almost incessant, and often so violent that I thought her actually in labour. She had a good deal of fever, and her strength failed so as to alarm me, and I determined, if possible, to hasten the delivery. On examination per vaginum, during a strong pain, I found the os tincæ dilated to the size of a dollar, but when the finger was placed against the membranes, no contraction of the longitudinal fibres could be felt—the action appeared to be confined entirely to the circular fibres. When the pain went off, the mouth of the uterus was fully relaxed. Repeated examinations gave the same result.

I ruptured the membranes, with the hope of inducing a different action, but was disappointed. I gave ergot, but without effect. The case finally became so urgent that I determined to open the head, which, with the assistance of Dr. Fearn, I did. All of the bones of the head were removed, and the child still did not advance. The uterus was in a very unfavourable condition for an operation of this kind, and the patient suffered so much, and was so much exhausted, that we thought it most prudent to desist for the present; and, after removing the head, we left her at midnight—about daylight she died—and when I went to see her about seven o'clock in the morning, I found her laid out in her burial clothes. About mid-day, I received a message that the child was born, and a request to visit her immediately. I did so, and found that a full grown fœtus had been expelled about six hours after death. She had become distended to fully twice her natural size with gas—not only the abdomen and thorax, but the head, neck, and extremities. I have no doubt that the child was forced out by this extrication of gas.—*Ibid.*

